# 2-(4-Chloro­phen­yl)-2,3-di­hydro­quinolin-4(1*H*)-one

**DOI:** 10.1107/S1600536814001548

**Published:** 2014-01-25

**Authors:** Meryem Chelghoum, Abdelmalek Bouraiou, Sofiane Bouacida, Mebarek Bahnous, Ali Belfaitah

**Affiliations:** a Laboratoire des Produits Naturels d’Origine Végétale et de Synthèse Organique, PHYSYNOR Université Constantine 1, 25000 Constantine, Algeria; b Unité de Recherche de Chemie de l’Environnement et Moléculaire Structurale, CHEMS, Université Constantine 1, 25000 , Algeria; c Département Sciences de la Matière, Faculté des Sciences Exactes et Sciences de la Nature et de la Vie, Université Oum El Bouaghi, 04000 Oum El Bouaghi, Algeria

## Abstract

The title mol­ecule, C_15_H_12_ClNO, features a di­hydro­quinolin-4(1*H*)-one moiety attached to a chloro­benzene ring. The heterocyclic ring has a half-chair conformation with the methine C atom lying 0.574 (3) Å above the plane of the five remaining atoms (r.m.s. deviation = 0.0240 Å). The dihedral angles between the terminal benzene rings is 77.53 (9)°, indicating a significant twist in the mol­ecule. In the crystal, supra­molecular zigzag chains along the *c-*axis direction are sustained by N—H⋯O hydrogen bonds. These are connected into double chains by C—H⋯π inter­actions.

## Related literature   

For background to and chemical reactivity of quinolone heterocycles, see: Diesbach & Kramer (1945 ▶); Prakash *et al.* (1994 ▶); Singh & Kapil (1993 ▶); Kalinin *et al.* (1992 ▶); Chauvin & Olivier (1996 ▶). For related structures, see: Bouraiou *et al.* (2008 ▶, 2011 ▶); Benzerka *et al.* (2011 ▶); Chelghoum *et al.* (2012 ▶).
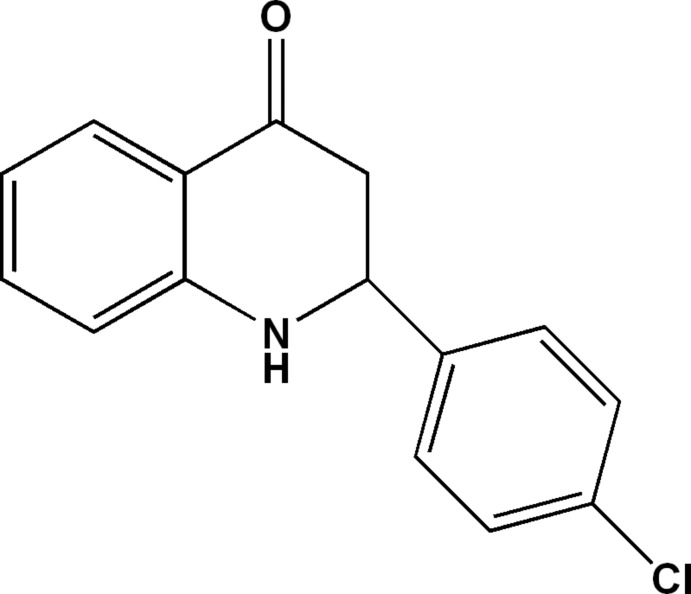


## Experimental   

### 

#### Crystal data  

C_15_H_12_ClNO
*M* *_r_* = 257.71
Monoclinic, 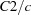

*a* = 17.703 (2) Å
*b* = 10.7537 (17) Å
*c* = 13.658 (2) Å
β = 105.486 (6)°
*V* = 2505.8 (6) Å^3^
*Z* = 8
Mo *K*α radiation
μ = 0.29 mm^−1^
*T* = 150 K
0.17 × 0.12 × 0.06 mm

#### Data collection  

Bruker APEXII diffractometer
Absorption correction: multi-scan (*SADABS*; Sheldrick, 2002 ▶) *T*_min_ = 0.932, *T*_max_ = 0.983
15688 measured reflections
2852 independent reflections
2314 reflections with *I* > 2σ(*I*)
*R*_int_ = 0.037

#### Refinement  

*R*[*F* ^2^ > 2σ(*F* ^2^)] = 0.045
*wR*(*F* ^2^) = 0.107
*S* = 1.08
2852 reflections
166 parameters
H atoms treated by a mixture of independent and constrained refinement
Δρ_max_ = 0.53 e Å^−3^
Δρ_min_ = −0.39 e Å^−3^

### 

Data collection: *SMART* (Bruker, 2001 ▶); cell refinement: *SAINT* (Bruker, 2001 ▶); data reduction: *SAINT*; program(s) used to solve structure: *SIR2002* (Burla *et al.*, 2005 ▶); program(s) used to refine structure: *SHELXL97* (Sheldrick, 2008 ▶); molecular graphics: *ORTEP-3 for Windows* (Farrugia, 2012 ▶) and *DIAMOND* (Brandenburg & Berndt, 2001 ▶); software used to prepare material for publication: *WinGX* (Farrugia, 2012 ▶).

## Supplementary Material

Crystal structure: contains datablock(s) I. DOI: 10.1107/S1600536814001548/tk5289sup1.cif

Structure factors: contains datablock(s) I. DOI: 10.1107/S1600536814001548/tk5289Isup2.hkl

Supporting information file. DOI: 10.1107/S1600536814001548/tk5289Isup3.cml

CCDC reference:

Additional supporting information:  crystallographic information; 3D view; checkCIF report

## Figures and Tables

**Table 1:** Hydrogen-bond geometry (Å, °) *Cg*2 and *Cg*3 are the centroids of the C1–C6 and C10–C15 benzene rings, respectively.

*D*—H⋯*A*	*D*—H	H⋯*A*	*D*⋯*A*	*D*—H⋯*A*
N1—H1*N*⋯O1^i^	0.84 (2)	2.15 (2)	2.957 (2)	162 (2)
C5—H5⋯*Cg*3^ii^	0.93	2.83	3.641 (2)	146
C11—H11⋯*Cg*2^iii^	0.93	2.63	3.465 (2)	149

Symmetry codes: (i) 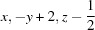; (ii) 
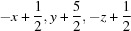
; (iii) 
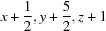
.

**Fig. 1.**
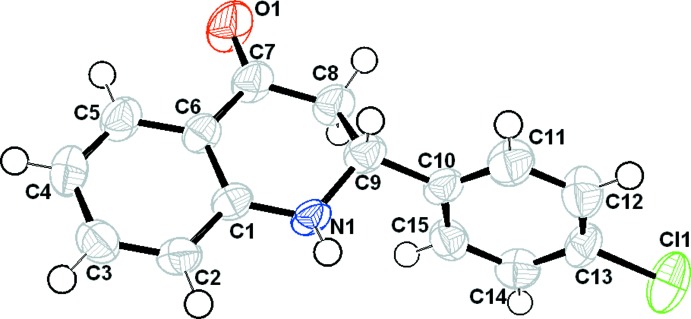
The molecular geometry of (I) with the atom-labelling scheme. Displacement ellipsoids are drawn at the 50% probability level. H atoms are represented as small spheres of arbitrary radius.

**Fig. 2.**
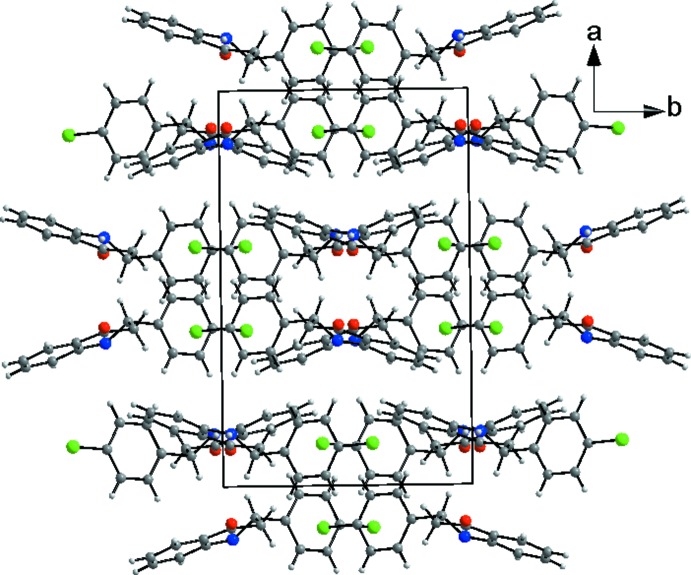
Alternating double layers parallel to (100) in (I), viewed down the c axis.

**Fig. 3.**
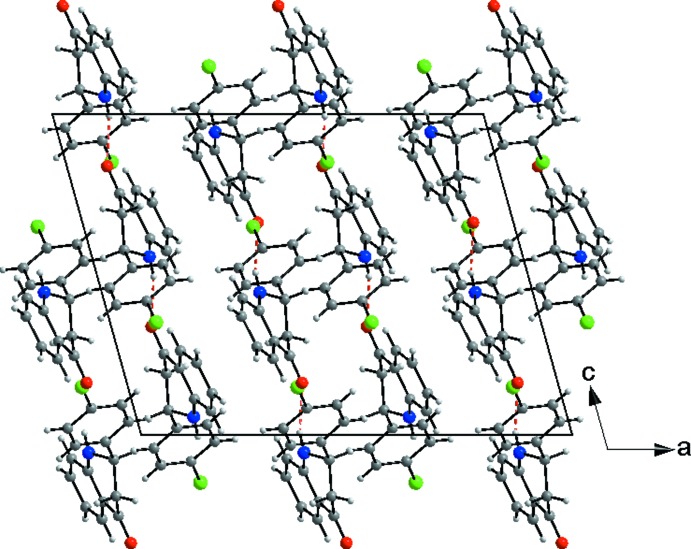
A diagram of the layered crystal packing of (I), viewed down the b axis showing hydrogen bonds as dashed lines.

## supplementary crystallographic information

### 2.1. Synthesis and crystallization

The corresponding 2'-amino­chalcone (0.5 mmol) and [bmim]BF_4_ (1 g) were heating at 150 °C for 2.5 h; bmim is butyl­methyl­imidazolium. The crude product was isolated by repeated extraction with di­ethyl ether (7×10 ml). Filtration of the residue through a silica plug gave the 2-(4-chloro­phenyl)-2,3-di­hydro­quinolin-4(1*H*)-one (I). Single crystals suitable for the X-ray diffraction analysis were obtained by dissolving the pure compound in an Et_2_O/CHCl_3_ mixture and allowing the solution to slowly evaporate at room temperature.

### 2.2. Refinement

The C-bound H atoms were geometrically placed (C—H = 0.93–0.98 Å) and refined as riding with *U*_iso_(H) = 1.2*U*_eq_(C). The H1N atom was refined with *U*_iso_(H) = 1.2*U*_eq_(N). Owing to poor agreement, the (1 1 0) reflection was omitted from the final cycles of refinement.

### 3. Results and discussion

2-Aryl­quinolo-4-ones are nitro­gen-containing analogues flavanones and flavones, and are characterized by a benzo ring fused to six-membered nitro­gen containing heterocyclic ring with an aryl substituent at position 2. The quinolone heterocyclic ring has many reactive sites for possible transformation and can also result in different degree of unsaturation (Diesbach & Kramer, 1945; Prakash *et al.*, 1994; Singh & Kapil, 1993; Kalinin *et al.*, 1992). To date, numerous accounts have been reported in the literature for the synthesis of quinolone, due to their frequent occurrence in biologically inter­esting molecules. RTILs have proven to be viable reaction media for numerous types of reaction, including, for example, Friedel–Crafts alkyl­ations, Diels–Alder, Knoevenagel, 1,3-dipolar cyclo­additions, and in three component coupling reactions (Chauvin & Olivier, 1996). As a part of our program directed toward the synthesis of new suitably functionalized heterocyclic compounds of potential biological activity (Bouraiou *et al.*, 2008, 2011; Benzerka *et al.*, 2011) and following our successes in the area of ionic liquid catalyzed 2-amino­chalones isomerization into the corresponding 2-phenyl-2,3-di­hydro­quinolin-4(1*H*)-one (Chelghoum *et al.*, 2012), we envisioned to get some information on the spatial arrangements of this type of compounds. We report herein the synthesis and single-crystal X-ray structure of 2-(4-chloro­phenyl)-2,3-di­hydro­quinolin-4(1*H*)-one (I). The molecular geometry and the atom-numbering scheme of (I) are shown in Fig. 1 and features a di­hydro­quinolin-4(1*H*)-one moiety attached to a chloro­benzene group. The crystal packing can be described as alternating double layers parallel to the (100) along the *a* axis (Fig. 2). It is stabilized by N—H···O hydrogen bonding and C—H···π inter­actions (Fig. 3; Table 1).

### Crystal data

**Table:**

C_15_H_12_ClNO	*F*(000) = 1072
*M**_r_* = 257.71	*D*_x_ = 1.366 Mg m^−^^3^
Monoclinic, *C*2/*c*	Mo *K*α radiation, λ = 0.71073 Å
Hall symbol: -C 2yc	Cell parameters from 5286 reflections
*a* = 17.703 (2) Å	θ = 2.4–27.2°
*b* = 10.7537 (17) Å	µ = 0.29 mm^−^^1^
*c* = 13.658 (2) Å	*T* = 150 K
β = 105.486 (6)°	Prism, colourless
*V* = 2505.8 (6) Å^3^	0.17 × 0.12 × 0.06 mm
*Z* = 8	

### Data collection

**Table:**

Bruker APEXII diffractometer	2314 reflections with *I* > 2σ(*I*)
Graphite monochromator	*R*_int_ = 0.037
CCD rotation images, thin slices scans	θ_max_ = 27.5°, θ_min_ = 2.5°
Absorption correction: multi-scan (*SADABS*; Sheldrick, 2002)	*h* = −22→21
*T*_min_ = 0.932, *T*_max_ = 0.983	*k* = −13→13
15688 measured reflections	*l* = −16→17
2852 independent reflections	

### Refinement

**Table:**

Refinement on *F*^2^	Primary atom site location: structure-invariant direct methods
Least-squares matrix: full	Secondary atom site location: difference Fourier map
*R*[*F*^2^ > 2σ(*F*^2^)] = 0.045	Hydrogen site location: inferred from neighbouring sites
*wR*(*F*^2^) = 0.107	H atoms treated by a mixture of independent and constrained refinement
*S* = 1.08	*w* = 1/[σ^2^(*F*_o_^2^) + (0.0328*P*)^2^ + 4.0966*P*] where *P* = (*F*_o_^2^ + 2*F*_c_^2^)/3
2852 reflections	(Δ/σ)_max_ = 0.001
166 parameters	Δρ_max_ = 0.53 e Å^−^^3^
0 restraints	Δρ_min_ = −0.39 e Å^−^^3^

### Special details

**Table:**

Geometry. All e.s.d.'s (except the e.s.d. in the dihedral angle between two l.s. planes) are estimated using the full covariance matrix. The cell e.s.d.'s are taken into account individually in the estimation of e.s.d.'s in distances, angles and torsion angles; correlations between e.s.d.'s in cell parameters are only used when they are defined by crystal symmetry. An approximate (isotropic) treatment of cell e.s.d.'s is used for estimating e.s.d.'s involving l.s. planes.
Refinement. Refinement of *F*^2^ against ALL reflections. The weighted *R*-factor *wR* and goodness of fit *S* are based on *F*^2^, conventional *R*-factors *R* are based on *F*, with *F* set to zero for negative *F*^2^. The threshold expression of *F*^2^ > σ(*F*^2^) is used only for calculating *R*-factors(gt) *etc*. and is not relevant to the choice of reflections for refinement. *R*-factors based on *F*^2^ are statistically about twice as large as those based on *F*, and *R*- factors based on ALL data will be even larger.

### Fractional atomic coordinates and isotropic or equivalent isotropic displacement parameters (Å^2^)

**Table:**

	*x*	*y*	*z*	*U*_iso_*/*U*_eq_	
C1	0.14660 (10)	1.07351 (16)	0.61189 (13)	0.0207 (4)	
C2	0.17699 (11)	1.17882 (17)	0.57430 (14)	0.0245 (4)	
H2	0.1876	1.1752	0.5112	0.029*	
C3	0.19115 (11)	1.28710 (18)	0.63005 (15)	0.0281 (4)	
H3	0.2118	1.3555	0.6044	0.034*	
C4	0.17487 (12)	1.29588 (18)	0.72502 (15)	0.0307 (4)	
H4	0.1839	1.3697	0.7618	0.037*	
C5	0.14538 (11)	1.19394 (18)	0.76280 (14)	0.0283 (4)	
H5	0.1343	1.1994	0.8255	0.034*	
C6	0.13150 (10)	1.08117 (16)	0.70848 (13)	0.0225 (4)	
C7	0.10478 (11)	0.97037 (18)	0.75251 (13)	0.0271 (4)	
C8	0.09756 (12)	0.85216 (18)	0.69154 (13)	0.0282 (4)	
H8A	0.0563	0.8016	0.7056	0.034*	
H8B	0.1462	0.806	0.7138	0.034*	
C9	0.07953 (11)	0.87276 (17)	0.57716 (13)	0.0256 (4)	
H9	0.0258	0.904	0.5523	0.031*	
C10	0.08698 (11)	0.75256 (16)	0.52145 (13)	0.0241 (4)	
C11	0.02108 (12)	0.69804 (18)	0.45834 (15)	0.0315 (4)	
H11	−0.0276	0.7351	0.4508	0.038*	
C12	0.02618 (12)	0.58890 (19)	0.40597 (16)	0.0329 (5)	
H12	−0.0186	0.5527	0.364	0.04*	
C13	0.09869 (11)	0.53526 (16)	0.41728 (14)	0.0264 (4)	
C14	0.16620 (11)	0.58739 (18)	0.47938 (14)	0.0277 (4)	
H14	0.2148	0.5505	0.4858	0.033*	
C15	0.15978 (11)	0.69599 (18)	0.53192 (14)	0.0274 (4)	
H15	0.2045	0.7314	0.5746	0.033*	
N1	0.13339 (9)	0.96526 (14)	0.55585 (11)	0.0224 (3)	
H1N	0.1307 (12)	0.9735 (19)	0.4941 (16)	0.027*	
O1	0.09352 (10)	0.96926 (14)	0.83773 (10)	0.0422 (4)	
Cl1	0.10656 (4)	0.39956 (5)	0.35097 (4)	0.04532 (18)	

### Atomic displacement parameters (Å^2^)

**Table:**

	*U*^11^	*U*^22^	*U*^33^	*U*^12^	*U*^13^	*U*^23^
C1	0.0211 (8)	0.0223 (9)	0.0191 (8)	0.0041 (7)	0.0061 (7)	0.0013 (7)
C2	0.0264 (9)	0.0259 (9)	0.0238 (9)	0.0020 (7)	0.0112 (7)	0.0023 (7)
C3	0.0274 (10)	0.0244 (9)	0.0338 (10)	−0.0023 (8)	0.0103 (8)	0.0022 (8)
C4	0.0348 (11)	0.0257 (10)	0.0300 (10)	−0.0025 (8)	0.0060 (8)	−0.0080 (8)
C5	0.0340 (11)	0.0308 (10)	0.0197 (9)	−0.0009 (8)	0.0068 (8)	−0.0045 (7)
C6	0.0258 (9)	0.0243 (9)	0.0168 (8)	0.0013 (7)	0.0048 (7)	−0.0003 (7)
C7	0.0368 (11)	0.0291 (10)	0.0161 (8)	−0.0002 (8)	0.0083 (7)	0.0010 (7)
C8	0.0415 (11)	0.0256 (9)	0.0203 (9)	−0.0012 (8)	0.0133 (8)	0.0025 (7)
C9	0.0315 (10)	0.0250 (9)	0.0224 (9)	0.0014 (7)	0.0106 (7)	0.0013 (7)
C10	0.0345 (10)	0.0206 (9)	0.0210 (8)	0.0002 (7)	0.0142 (7)	0.0013 (7)
C11	0.0282 (10)	0.0289 (10)	0.0377 (11)	0.0059 (8)	0.0092 (8)	−0.0023 (8)
C12	0.0286 (10)	0.0291 (10)	0.0370 (11)	0.0013 (8)	0.0017 (8)	−0.0054 (8)
C13	0.0367 (10)	0.0182 (9)	0.0253 (9)	0.0036 (7)	0.0098 (8)	−0.0026 (7)
C14	0.0260 (9)	0.0271 (10)	0.0305 (10)	0.0054 (8)	0.0085 (8)	0.0040 (8)
C15	0.0272 (10)	0.0296 (10)	0.0244 (9)	−0.0061 (8)	0.0049 (7)	−0.0003 (7)
N1	0.0332 (8)	0.0215 (8)	0.0154 (7)	0.0008 (6)	0.0118 (6)	0.0012 (6)
O1	0.0737 (11)	0.0384 (8)	0.0198 (7)	−0.0086 (8)	0.0219 (7)	−0.0018 (6)
Cl1	0.0608 (4)	0.0271 (3)	0.0468 (3)	0.0075 (2)	0.0122 (3)	−0.0135 (2)

### Geometric parameters (Å, º)

**Table:**

C1—N1	1.378 (2)	C8—H8B	0.97
C1—C2	1.408 (2)	C9—N1	1.460 (2)
C1—C6	1.417 (2)	C9—C10	1.523 (2)
C2—C3	1.377 (3)	C9—H9	0.98
C2—H2	0.93	C10—C11	1.382 (3)
C3—C4	1.405 (3)	C10—C15	1.398 (3)
C3—H3	0.93	C11—C12	1.390 (3)
C4—C5	1.373 (3)	C11—H11	0.93
C4—H4	0.93	C12—C13	1.378 (3)
C5—C6	1.409 (2)	C12—H12	0.93
C5—H5	0.93	C13—C14	1.386 (3)
C6—C7	1.469 (3)	C13—Cl1	1.7425 (18)
C7—O1	1.232 (2)	C14—C15	1.391 (3)
C7—C8	1.506 (3)	C14—H14	0.93
C8—C9	1.525 (2)	C15—H15	0.93
C8—H8A	0.97	N1—H1N	0.84 (2)
			
N1—C1—C2	120.10 (15)	N1—C9—C10	109.29 (14)
N1—C1—C6	121.33 (15)	N1—C9—C8	109.52 (15)
C2—C1—C6	118.56 (16)	C10—C9—C8	111.48 (15)
C3—C2—C1	120.62 (16)	N1—C9—H9	108.8
C3—C2—H2	119.7	C10—C9—H9	108.8
C1—C2—H2	119.7	C8—C9—H9	108.8
C2—C3—C4	121.02 (17)	C11—C10—C15	118.75 (17)
C2—C3—H3	119.5	C11—C10—C9	120.02 (17)
C4—C3—H3	119.5	C15—C10—C9	121.23 (17)
C5—C4—C3	119.06 (17)	C10—C11—C12	121.28 (18)
C5—C4—H4	120.5	C10—C11—H11	119.4
C3—C4—H4	120.5	C12—C11—H11	119.4
C4—C5—C6	121.32 (17)	C13—C12—C11	118.85 (18)
C4—C5—H5	119.3	C13—C12—H12	120.6
C6—C5—H5	119.3	C11—C12—H12	120.6
C5—C6—C1	119.40 (16)	C12—C13—C14	121.64 (17)
C5—C6—C7	120.84 (16)	C12—C13—Cl1	119.59 (15)
C1—C6—C7	119.71 (16)	C14—C13—Cl1	118.77 (15)
O1—C7—C6	123.07 (17)	C13—C14—C15	118.64 (17)
O1—C7—C8	120.19 (17)	C13—C14—H14	120.7
C6—C7—C8	116.57 (15)	C15—C14—H14	120.7
C7—C8—C9	114.05 (15)	C14—C15—C10	120.85 (17)
C7—C8—H8A	108.7	C14—C15—H15	119.6
C9—C8—H8A	108.7	C10—C15—H15	119.6
C7—C8—H8B	108.7	C1—N1—C9	119.19 (14)
C9—C8—H8B	108.7	C1—N1—H1N	115.2 (15)
H8A—C8—H8B	107.6	C9—N1—H1N	114.2 (14)
			
N1—C1—C2—C3	−179.37 (16)	N1—C9—C10—C11	−126.55 (18)
C6—C1—C2—C3	−0.5 (3)	C8—C9—C10—C11	112.2 (2)
C1—C2—C3—C4	−0.7 (3)	N1—C9—C10—C15	52.8 (2)
C2—C3—C4—C5	0.9 (3)	C8—C9—C10—C15	−68.4 (2)
C3—C4—C5—C6	0.2 (3)	C15—C10—C11—C12	0.1 (3)
C4—C5—C6—C1	−1.4 (3)	C9—C10—C11—C12	179.52 (18)
C4—C5—C6—C7	176.00 (18)	C10—C11—C12—C13	−0.4 (3)
N1—C1—C6—C5	−179.59 (16)	C11—C12—C13—C14	0.0 (3)
C2—C1—C6—C5	1.5 (3)	C11—C12—C13—Cl1	−179.13 (15)
N1—C1—C6—C7	3.0 (3)	C12—C13—C14—C15	0.6 (3)
C2—C1—C6—C7	−175.94 (16)	Cl1—C13—C14—C15	179.75 (14)
C5—C6—C7—O1	−0.3 (3)	C13—C14—C15—C10	−0.8 (3)
C1—C6—C7—O1	177.09 (18)	C11—C10—C15—C14	0.5 (3)
C5—C6—C7—C8	−175.52 (17)	C9—C10—C15—C14	−178.88 (16)
C1—C6—C7—C8	1.9 (3)	C2—C1—N1—C9	−159.94 (16)
O1—C7—C8—C9	156.11 (19)	C6—C1—N1—C9	21.2 (2)
C6—C7—C8—C9	−28.6 (2)	C10—C9—N1—C1	−168.81 (15)
C7—C8—C9—N1	49.0 (2)	C8—C9—N1—C1	−46.4 (2)
C7—C8—C9—C10	170.10 (16)		

### Hydrogen-bond geometry (Å, º)

Cg2 and Cg3 are the centroids of the C1–C6 and C10–C15 benzene rings, respectively.

**Table:**

*D*—H···*A*	*D*—H	H···*A*	*D*···*A*	*D*—H···*A*
N1—H1*N*···O1^i^	0.84 (2)	2.15 (2)	2.957 (2)	162 (2)
C5—H5···*Cg*3^ii^	0.93	2.83	3.641 (2)	146
C11—H11···*Cg*2^iii^	0.93	2.63	3.465 (2)	149

Symmetry codes: (i) *x*, −*y*+2, *z*−1/2; (ii) −*x*+1/2, *y*+5/2, −*z*+1/2; (iii) *x*+1/2, *y*+5/2, *z*+1.
